# Prevalence, awareness, and treatment of depression among community-dwelling stroke survivors in Korea

**DOI:** 10.1038/s41598-022-08126-y

**Published:** 2022-03-08

**Authors:** Eung-Joon Lee, Oh Deog Kwon, Seung Jae Kim

**Affiliations:** 1 Department of Neurology, Seoul National University Hospital, Seoul, Republic of Korea; 2 Instituite of Public Health and Medical Care, Seoul National University Hospital, Seoul, Republic of Korea; 3 Movinci Clinic, Seoul, Republic of Korea; 4 International Healthcare Center, Seoul St. Mary’s Hospital, College of Medicine, The Catholic University of Korea, 222 Banpo-daero, Seocho-gu, Seoul, 06591 Republic of Korea; 5 Department of Family Medicine, College of Medicine, The Catholic University of Korea, Seoul, Republic of Korea

**Keywords:** Stroke, Depression, Epidemiology

## Abstract

Post-stroke depression (PSD), a prevalent complication of stroke, causes poor outcomes. However, little is known about its prevalence and management among community-dwelling stroke survivors. Thus, we investigated the prevalence, awareness, and treatment of PSD in a community setting. A cross-sectional study was performed using representative data from the Korea National Health and Nutrition Surveys 2014, 2016, and 2018. A total of 11,122 participants aged ≥ 40 years were categorized, including 343 stroke survivors and 10,779 non-stroke survivors. We then calculated and compared the prevalence, awareness (formal diagnosis of depression by a doctor), and treatment rates of depression between the two groups. Depression was defined as a score ≥ 10 in the nine-item Patient Health Questionnaire (PHQ-9). Depression was significantly more prevalent among stroke survivors than in non-stroke survivors (22.2% vs. 8.5%, respectively), while the differences in the awareness and treatment rates were insignificant. However, only 46.8% of stroke survivors with PSD were aware of their condition, and only 20.5% were receiving treatment. These results suggest that clinicians should more actively screen for and treat depression among stroke survivors.

## Introduction

Stroke remains the leading cause of death worldwide and in Korea^1,2^. Its overall burden is also high as it is one of the top-ranked causes of disability-adjusted life-years^3^. Depression is one of the most prevalent sequelae of stroke, with about one-third of stroke survivors developing depression at any time-point within 5 years after the stroke event^4,5^. Furthermore, various studies have reported that the presence of post-stroke depression (PSD) negatively affected the clinical outcomes of stroke survivors^4^. In fact, PSD is a well-known predictor of poorer functional outcomes^6,7^, quality of life^8,9^, life satisfaction^10^ and most importantly, higher mortality^11,12^. However, PSD is often underdiagnosed and undertreated, despite its high prevalence and influence on the post-stroke prognosis^13,14^. Although several previous studies have investigated the prevalence of PSD, these mainly were focused on the development of depression among hospital-based patients shortly after the stroke onset rather than the overall depression status of stroke survivors in a community setting^5,15^. In addition, few studies have examined the awareness and treatment rates of depression among stroke survivors, leading to little knowledge about these, especially in Korea. Therefore, this study aimed to assess the prevalence, awareness, and treatment of depression among community-dwelling stroke survivors using nationwide representative cross-sectional data from the Korea National Health and Nutrition Survey (KNHANES) to offer constructive perspectives in the screening and management of depression among stroke survivors.

## Results

### Baseline characteristics

The baseline characteristics of the stroke and non-stroke survivors are presented in Table 1. In terms of socio-demographics, stroke survivors were significantly older than non-stroke survivors, with 64.3% of stroke survivors being 60 years and older and 65.8% of non-stroke survivors being younger than 60 years. Stroke survivors were also more likely to be without spouses (66.6% vs. 18.5%, respectively), unemployed (66.6% vs. 34.6%, respectively), less educated (middle school or lower: 61.9% vs. 31.7%, respectively; high school: 27.4% vs. 34.5%, respectively; and college or higher 10.7% vs. 33.8%, respectively), have lower income (low or lower middle-class income: 69.3% vs. 41.2%, respectively), reside in urban areas (41.9% vs. 35.2%, respectively), and be Medicaid beneficiaries (12.8% vs. 3.8%, respectively). Regarding health status, stroke survivors had a significantly higher percentage of individuals with a PHQ-9 score ≥ 10 (16.5% vs. 6.7%, respectively) and a 20–27 PHQ-9 score (3.5% vs. 0.5%, respectively). Furthermore, limitations in daily activities (68.4% vs. 7.8%), poorer self-rated health (poor: 51.6% vs. 19.2%, fair: 39.2% vs. 52.6%, and good: 9.2% vs. 28.2%), and comorbidities (72.7% vs. 37.0%) were significantly more common in stroke survivors than in non-stroke survivors. Moreover, more stroke survivors were overweight or obese compared to non-stroke survivors (66.7% vs. 51.1%, respectively), but the difference was not significant. In terms of the specific comorbidities, a significantly higher percentage of stroke survivors had hypertension (65.2% vs. 26.9%), DM (29.9% vs. 10.4%), and ischemic heart disease (12.1% vs. 2.9%), while the differences in the prevalence of other comorbidities were insignificant. Lastly, in terms of the participants’ health behaviors, stroke survivors were less likely to sufficiently engage in physical activity (36.5% vs. 44.9%, respectively) and undergo regular health check-ups (63.8% vs. 73.9%, respectively). Stroke survivors also had a lower percentage of current smokers and risky drinkers than non-stroke survivors, though the differences were insignificant.

**Table 1:** Baseline characteristics of non-stroke survivors and stroke survivors.

Characteristics	Non-stroke survivors (n = 10,779) % (SE)	Stroke survivors (n = 343) % (SE)	χ^2^	*P* value
**Sociodemographics**				
Sex			3.484	0.074
Male	48.1 (0.4)	53.7 (3.1)		
Female	51.9 (0.4)	46.3 (3.1)		
Age (years)			229.876	0.000
40–49	33.6 (0.7)	6.7 (1.9)		
50–59	32.2 (0.7)	21.2 (2.9)		
60–69	19.8 (0.5)	29.9 (2.9)		
70–79	11.9 (0.4)	32.7 (2.9)		
≥ 80	2.5 (0.2)	9.5 (1.7)		
Marital status			39.765	0.000
Married	81.5 (0.6)	33.4 (3.2)		
Single/divorced/seperated/widowed	18.5 (0.6)	66.6 (3.2)		
Emplyoment status			122.524	0.000
Employed	65.4 (0.6)	33.4 (3.2)		
Unemployed	34.6 (0.6)	66.6 (3.2)		
Education status			124.329	0.000
Middle school or lower	31.7 (0.8)	61.9 (3.2)		
High school	34.5 (0.7)	27.4 (3.1)		
College or higher	33.8 (1.0)	10.7 (1.9)		
Household Income			157.377	0.000
Low	17.5 (0.7)	45.3 (3.2)		
Lower middle	23.7 (0.7)	24.0 (2.8)		
Upper middle	28.4 (0.7)	18.7 (2.7)		
High	30.4 (1.0)	11.9 (2.3)		
Residential area			5.319	0.045
Urban	35.2 (1.3)	41.9 (3.5)		
Rural	64.8 (1.3)	58.1 (3.5)		
Health insurance			58.312	0.000
Medicaid	3.8 (0.3)	12.8 (2.2)		
National Health insurance	96.2 (0.3)	87.2 (2.2)		
**Health-status**				
PHQ-9 score			91.825	0.000
< 10	95.3 (0.3)	83.5 (2.5)		
10–19	4.2 (0.2)	13.0 (2.3)		
20–27	0.5 (0.1)	3.5 (1.4)		
BMI (kg/m^2^)			3.763	0.330
< 18.5	2.4 (0.2)	1.9 (0.7)		
18.5–22.9	36.4 (0.6)	31.4 (3.0)		
23–24.9	24.9 (0.5)	27.8 (3.0)		
≥ 25	26.2 (0.6)	38.9 (3.1)		
Comorbidities			148.120	0.000
No	63.0 (0.6)	27.3 (2.8)		
Yes	37.0 (0.6)	72.7 (2.8)		
Hypertension	26.9 (0.5)	65.2 (3.0)	199.831	0.000
Diabetes mellitus	10.4 (0.3)	29.9 (2.8)	96.990	0.000
Ischemic heart disease	2.9 (0.2)	12.1 (2.1)	76.218	0.000
Cancer*	6.0 (0.3)	4.5 (1.2)	1.081	0.265
Chronic pulmonary diseases^†^	3.2 (0.2)	4.5 (1.3)	1.401	0.258
Liver cirrhosis	0.4 (0.1)	0.5 (0.4)	0.306	0.837
Chronic renal disease	0.4 (0.1)	0.6 (0.6)	0.524	0.535
Self-rated health			0.000	0.000
Poor	19.2 (0.5)	51.6 (3.2)		
Fair	52.6 (0.5)	39.2 (3.0)		
Good	28.2 (0.5)	9.2 (1.9)		
Limitation in daily acitivities			189.493	0.000
Yes	7.8 (0.3)	68.4 (3.1)		
No	92.2 (0.3)	31.6 (3.1)		
**Health behavior**				
Smoking status			0.241	0.653
Never/past smoker	79.8 (0.5)	81.0 (2.5)		
Current smoker	20.2 (0.5)	19.0 (2.5)		
Drinking status			1.956	0.277
Non-risky drinking	87.4 (0.4)	90.2 (2.3)		
Risky drinking	12.6 (0.4)	9.8 (2.3)		
Physical activity			7.862	0.010
Sufficient	44.9 (0.6)	36.5 (3.1)		
Insufficient	55.1 (0.6)	63.5 (3.1)		
Health screening within the last 2 years			14.499	0.000
Yes	73.9 (0.5)	63.8 (2.9)		
No	26.1 (0.5)	36.2 (2.9)		

All data were weighted to the Korean standard population.

*P* values were obtained by chi-square test.

*Stomach, liver, colon, breast, cervix, lung and thyroid cancer.

^†^Asthma and chronic obstructive pulmonary disease.

SE, standard error; SD, standard deviation; PHQ-9, The nine-item Patient Health Questionnaire; BMI, body mass index; LDL-C, low density lipoprotein cholesterol; FBS. Fasting blood sugar.

### Prevalence, awareness, and treatment of depression between stroke and non-stroke survivors

The prevalence of depression was significantly higher among stroke survivors than among non-stroke survivors (22.2% vs. 8.5%, respectively). Among those with depression, non-stroke survivors had higher awareness (59.3% vs. 46.8%, respectively) and treatment (22.3% vs. 20.5%, respectively) rates, but the difference between the two groups was statistically insignificant (Table 2).

**Table 2:** Comparison of prevalence, awareness, and treatment of depression between non-stroke survivors and stroke survivors.

Variables	Non-stroke survivors		Stroke survivors		*P* value
	N*	% (SE)^†^	N*	% (SE)^†^	
All participants	10,779	100 (0.0)	343	100 (0.0)	
Prevalence	1005	8.5 (0.3)	75	22.2 (2.7)	0.000
**Participant with depression**					
Awareness	569	59.3 (1.9)	36	46.8 (6.5)	0.585
Treatment	222	22.3 (1.6)	19	20.5 (5.2)	0.740

*Unweighted raw data numbers.

^†^Weighted to the Korean standard population.

*P* values were obtained by chi-square test.

## Discussion

In this nationwide population-based survey, depression was significantly more prevalent among stroke survivors than in non-stroke survivors (22.2% vs. 8.5%, respectively). This trend was consistent with previous studies as depression was more common in patients with stroke, and stroke was independently associated with an increased risk of developing depression^16,17^. Thus, careful attention and close monitoring of the potential development of depression are continuously required for stroke survivors.

The prevalence rate of depression among stroke survivors (22.2%) is somewhat lower than the rate reported by previous meta-analyses that investigated the prevalence of PSD (30%)^5,18^. This discrepancy may be due to the differences in the participants’ settings. Most of the studies in the meta-analyses were conducted among hospital-based patients with acute stroke and the frequency of PSD was assessed within 1–5 years of initial stroke onset. In contrast, our study examined the prevalence of depression among community-dwelling stroke survivors in the chronic phase of stroke. Hospital-based patients in the acute phase of stroke usually have higher rates of PSD than community-based patients because they are in more critical and severe conditions^19^. In addition, most PSDs are known to first occur within a year of stroke onset^20^. A study based in the United States, which investigated the frequency of PSD in a community setting using the National Health and Nutrition Examination Survey, also reported a lower prevalence of PSD than previous meta-analyses (17%)^21^. In the said study, the prevalence of PSD was defined solely with a PHQ score ≥ 10, whereas our study included a PHQ score ≥ 10, a formal diagnosis of depression by a doctor, or if the patient was currently being treated for depression. In fact, the prevalence rate was generally similar between the two studies when PSD was assessed using the same definition (16.5% vs 17%).

Although depression was notably more prevalent in stroke survivors than in non-stroke survivors, our study revealed that only 46.8% of the stroke survivors with depression were aware of their condition, and only 20.5% were undergoing treatment. These numbers are quite concerning considering that PSD has been established as a major risk factor for poor clinical outcomes, including increased risk of mortality among stroke survivors. To the best of our knowledge, this study was the first to determine the awareness and treatment rates of depression among stroke survivors. Although several studies have mentioned that PSD was often underdiagnosed and undertreated, none reported the actual rates of awareness and treatment of PSD^13,14^. These low rates might be due to social stigma in Korea toward people with mental illnesses. In many Asian cultures, including Korea, mental illness is viewed as a sign of personal weakness or insufficient willpower^22,23^. Thus, Koreans with a mental illness, especially the elderly, are reluctant to seek mental health services^22^. Furthermore, disorders of self-awareness are commonly found in patients who suffered stroke^24^. It has been reported that approximately 30% of stroke survivors are known to have vascular cognitive impairment or post-stroke dementia^25–27^ and having PSD itself correlated with impairment cognitive function such as memory, nonverbal problem solving, and attention and psychomotor speed^28^. Hence, this may be the reason for lower awareness and treatment of depression in stroke survivors than non-stroke survivors since those with impairment cognitive function would less likely to be aware of their depressive mood and treated for depression as a consequence. In addition, previous studies have identified that treating PSD with antidepressants not only improved the depressive symptoms but also survival^29,30^. Therefore, clinicians should be more active in detecting and treating depression among stroke survivors.

Our study had several limitations that need to be addressed. First, recall bias was possible since the KNHANES data is based on a self-administered questionnaire. Second, the details of stroke, including its type (ischemic or hemorrhagic), severity, and location, could not be included in the analysis because the KNHANES does not collect this kind of information. Third, the participants’ premorbid status, including the history of depression before the diagnosis of stroke, could not be assessed due to its unavailability in the KNHANES. Fourth, the term PSD might not be appropriate for some participants since we could not fully assess the timeline of occurrence of stroke and depression of participants due to lack of information in the KNHANES data. However, we used the term PSD because there is no consensus on the definition of PSD regarding the timing of developing depression after stroke^5,31^. Fifth, the term awareness of depression could incorporate a wider breadth of meaning than just a formal diagnosis. Nevertheless, “diagnosis by a doctor” is the most commonly used definition when measuring awareness of notable chronic disease (e.g. hypertension, diabetes mellitus, dyslipidemia) using nationwide survey data like the KNHANES^32–34^. Some may argue that this definition is not suitable for mental diseases such as depression since those with PHQ score ≥ 10 have already self-reported at least some level of awareness of mood symptoms. However, there is a difference between being somewhat aware of depressive mood and recognizing it as a medical problem. Also, considering the fact that PSDs are frequently underdiagnosed and undertreated despite its significant influence on the later outcomes of stroke survivors^13,14^, we reckon that discovering stroke survivors who do not seek to see a doctor despite being aware of depressive mood to some degree would be very important from a public health point of view. For these reasons, we applied the stricter and narrower standard when defining the term “awareness” of depression. Lastly, this study only included Koreans with a relatively small sample size and missing values were handled with list-wise deletion; hence, the results of this study should be treated with caution and further studies with a larger sample which includes multiethnic populations should be conducted to verify our results. Despite these limitations, the objective estimation of the prevalence and management status of PSD in Korea using nationally representative KNHANES data constitutes this study’s major strength. We believe that our results provide valuable epidemiological information on PSD among community-dwelling stroke survivors.

## Conclusions

In this nationwide cross-sectional study, depression was significantly more prevalent among community-dwelling stroke survivors than in non-stroke survivors.

However, more than half of the patients with PSD were unaware of their condition, and approximately 80% of them were untreated. Thus, clinicians should more actively screen for and treat depression among stroke survivors.

## Methods

### Data source and study population

**Figure 1:**
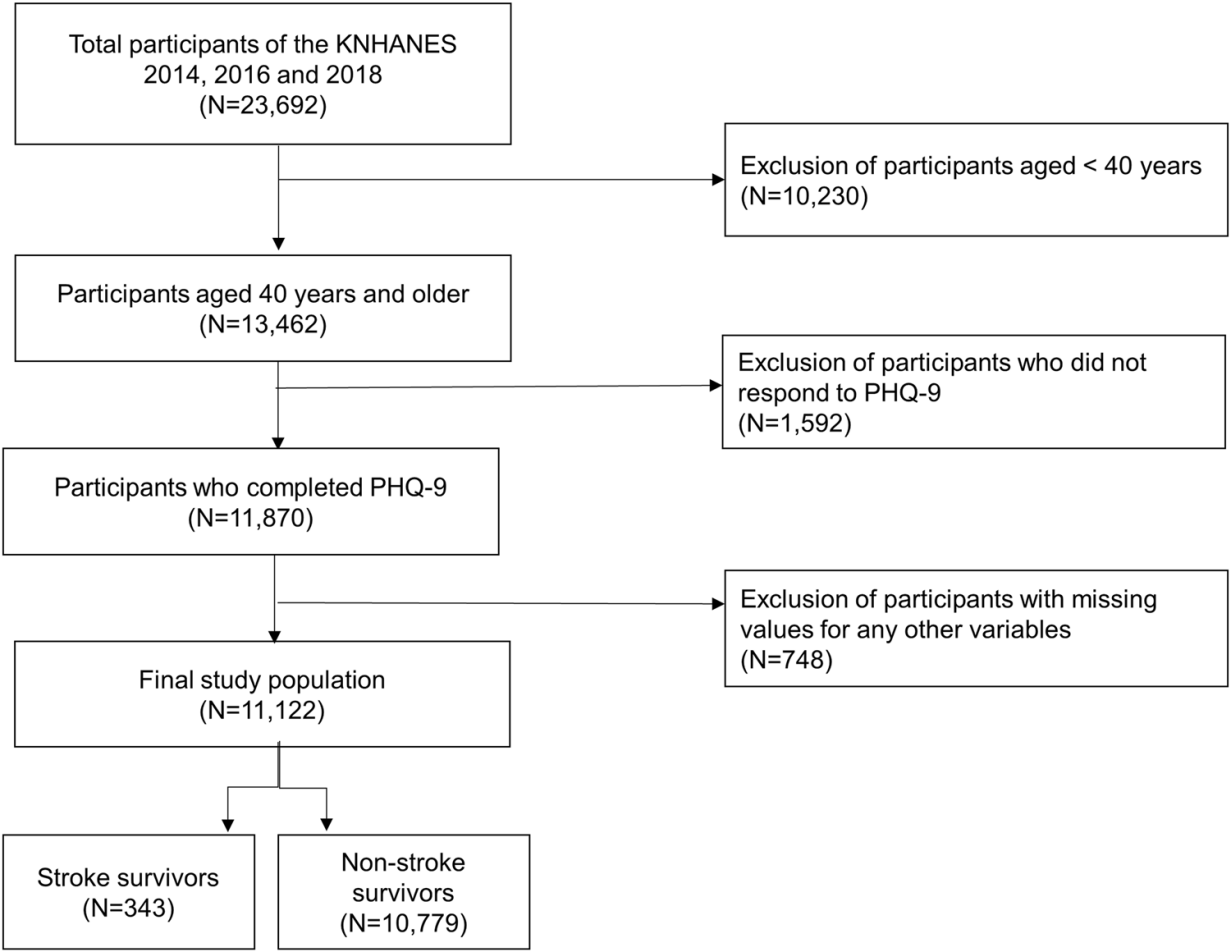
Selection process of study population. KNHANES, Korea National Health and Nutrition Examination Survey.

The KNHANES, an annual national surveillance program organized by the Korea Centers for Disease Control and Prevention since 1998, gathers extensive information on the non-institutionalized civilian population of Korea, including socio-demographics, general health status, and health behaviors, through personal interviews, direct physical examinations, and laboratory and clinical tests. The participants of the KNHANES were selected based on a rolling sampling design involving a complex, stratified, multistage, probability cluster survey to obtain unbiased and nationally representative data. The representativeness, validity, and other details of the KNHANES have been reviewed in previous studies^35,36^. This study analyzed data from the KNHANES 2014, 2016, and 2018 since the nine-item Patient Health Questionnaire (PHQ-9) was included in these iterations. Out of the 23,692 participants in the KNHANES 2014, 2016, and 2018, those aged 40 years and older were selected (N = 13,462). Participants who did not respond to PHQ-9 were excluded (N = 1592). List-wise deletion was applied to exclude participants with missing data (N = 748), resulting a total study population of 11,122 individuals. We then divided the participants into two groups according to their history of stroke. Stroke survivors (N = 343) were defined as participants who positively responded to the question asking for a prior diagnosis of stroke by a doctor, whereas those who replied negatively to the same question were considered as non-stroke survivors (N = 10,779) (Fig. 1). The present study was approved by the Institutional Review Board of Seoul St. Mary’s Hospital, Catholic University of Korea (Approval number: KC21ZASI0264). The study protocol conformed to the ethical guidelines of the 1975 Declaration of Helsinki. The requirement for written informed consent was waived since all data were fully anonymized and de-identified. The waiver was approved by the Institutional Review Board mentioned above.

### Outcome variables

The prevalence of depression was defined as the proportion of participants with a PHQ-9 score ≥ 10, a positive response to a formal diagnosis of depression by a doctor, or if the patient was currently receiving treatment for depression. The PHQ-9, the most commonly used screening tool for depression in primary care and other clinical settings^37^, consists of nine items that assess the frequency of depressive symptoms. The total PHQ-9 score can range from 0 to 27, with higher scores indicating more severe depression^19^. The standard cut-off score for detecting major depression is ten or higher. The accuracy, reliability, and validity of the said test in detecting and assessing the severity of depression have been well established^37,38^. Among the patients with depression, awareness of depression was defined as a positive response to a formal diagnosis of depression by a doctor, while treatment of depression was defined as those who responded positively to a question asking whether the participant was currently receiving treatment for depression.

### Other variables

We categorized the variables for baseline characteristics of participants into three dimensions: socio-demographic, health status, and health behavior. The socio-demographic dimension consisted of age, sex, marital status, employment status, household income, residential area, and type of medical insurance. The health status dimension included the status of obesity, comorbidities, self-rated health, and limitations in daily activities. Lastly, the health behavior dimension comprised the status of smoking, drinking, physical activity, and regular health screening. Each variable was gathered based on a self-reporting questionnaire of the KNHANES Health Interview Survey. Depression status was categorized based on the PHQ-9 cut-off scores for detecting and assessing the severity of depression (no depression: < 10, moderate depression: 10–19, and severe depression: 20–27)^39^. Obesity status was assessed by calculating the body mass index (m^2^/kg) and classifying it according to the Korean obesity standards (underweight: < 18.5 kg/m^2^, ideal weight: 18.5–22.9 kg/ m^2^, overweight: 23–24.9 kg/m^2^, and obese: ≥ 25.0 kg/m^2^)^40^. The presence of comorbidity was defined as a positive response to a formal diagnosis of any of the following conditions by a doctor: hypertension, diabetes mellitus, ischemic heart disease (myocardial infarction and angina), cancers (stomach, liver, breast, lung, thyroid, and other cancers), chronic pulmonary diseases (chronic obstructive pulmonary disease and asthma), liver cirrhosis, and chronic renal disease were included as comorbidities. Participants were classified as either current smokers or non-smokers (never or past smokers), according to their smoking status. For the drinking status, participants were categorized into risky drinkers (those who drank ≥ 2 times/week, with an average of ≥ 5 drinks/occasion for men and ≥ 7 drinks/occasion for women) and non-risky drinkers (those who drank less alcohol than risky drinkers)^41^. The physical activity status of participants was classified according to the Physical Activity Guidelines for Americans 2nd edition^42^, which recommends that adults engage in at least 150 min of moderate-intensity aerobic physical activity, 75 min of vigorous-intensity aerobic physical activity, or an equivalent combination of moderate and vigorous-intensity activity. Participants whose self-reported physical activity fulfilled these criteria were classified into the sufficient physical activity group. Regular health screening was defined as a positive response to having undergone a health check-up in the past 2 years.

### Statistical analyses

The survey sample weights recommended by the Korea Centers for Disease Control and Prevention were applied to all the analyses to produce estimates that represented the entire Korean population without bias^35^. Descriptive analyses were conducted to describe the baseline characteristics of the study population. The chi-square test was used to analyze the statistical differences in the baseline characteristics and the prevalence, awareness, and treatment rates of depression between the stroke and non-stroke survivors. All statistical analyses were conducted using STATA version 14.1 (Stata Corp., College Station, TX, USA). Statistical significance was defined as a two-sided *p* value < 0.05.
